# A Measles Outbreak in Riyadh in 2023: Clinical and Epidemiological Characteristics

**DOI:** 10.7759/cureus.48171

**Published:** 2023-11-02

**Authors:** Nourah Alruqaie, Bedoor Al Qadrah, Shahad Almansour, Eman Alghamdi, Musaed Alharbi

**Affiliations:** 1 Pediatric Infectious Diseases, King Abdulaziz Medical City, Riyadh, SAU; 2 Pediatrics, King Abdulaziz Medical City, Riyadh, SAU; 3 Public Health, King Abdulaziz Medical City, Riyadh, SAU

**Keywords:** saudi arabia, riyadh, epidemiology, vaccination, outbreak, measles

## Abstract

Background

Measles is a highly contagious viral disease that has recently made headlines due to outbreaks in several parts of the world. The disease can cause serious health complications, especially in young children, which has led to concerns about vaccination rates and public health policies. This study aims to investigate and describe the epidemiology, clinical characteristics, and outcomes of measles infection among children in Riyadh.

Methodology

We conducted a descriptive cross-sectional study among all pediatric patients with confirmed measles infection at a tertiary hospital from January 15, 2023, to March 15, 2023. We collected data including demographic characteristics, clinical presentations, and clinical outcomes.

Results

A total of 63 confirmed measles cases were reported. Most patients were under four years of age (82.7%), and 85.7% were unvaccinated. Adenovirus was the most common viral coinfection (12.7%). The most common complication was pneumonia (58.7%). Chest X-ray findings reported a localized right parenchymal infiltrate in 19% of patients and a patchy bilateral infiltrate in 15.9% of patients. In addition, 88.9% required hospital admission secondary to dehydration (47.6%) and hypoxia (41.3%). Among admitted patients, 17.5% were admitted to the pediatric intensive care unit (PICU), 9.5% were admitted due to respiratory failure, and 6.3% due to septic shock. Children under one year of age had a higher risk for PICU admission (p < 0.05). The mortality rate was 1.6%.

Conclusions

Measles is a serious disease that causes significant health effects and incurs high financial costs for public health systems. Vaccination remains the most effective way to prevent measles outbreaks and reduce their impact on individuals and communities.

## Introduction

The measles virus is a highly contagious and serious infectious disease spread by airborne respiratory droplets. Infected patients experience fever, conjunctivitis or coryza, cough, and a generalized rash that spreads craniocaudally. There is a high probability that the vast majority of patients recover from the disease after the pathogens are eliminated and they develop lifelong specific immunity preventing reinfection. The majority of patients recover without any complications, but approximately 30% of cases are complicated by diarrhea, pneumonia, acute otitis media, encephalitis, or even death. Infants, pre-schoolers, and toddlers are more likely to develop an unfavorable disease course. When a measles infection occurs naturally, in 0.01% of cases, it remains in the neural tissues and becomes dormant for several years, before progressing to subacute sclerosing panencephalitis (SSPE). SSPE is a debilitating encephalitis affecting children who were infected before the age of two years. Although partial and temporary remissions are possible, the disease is always fatal regardless of the patient’s age. Therefore, a reduction in disease burden is only possible through active immunization. Before vaccination, more than 90% of children were infected with the measles virus, resulting in more than 2 million deaths annually. In the post-vaccination era, measles cases decreased significantly as active immunization became available [1,2].

Between 2000 and 2016, the incidence of measles worldwide decreased by 84%. However, despite the success of active immunization programs in decreasing the incidence of measles and its related complications, several outbreaks have affected children and adults globally, with reported severe and deadly consequences [1-7]. The World Health Organization (WHO) has recently reported a surge in measles cases in multiple countries [1]. With an estimated surge of 556%, reaching nearly 870,000 cases, this is the highest number since 1996 [4].

In Saudi Arabia, several outbreaks have been reported in different regions [5-10]. AlJowf, AlQassim, Jeddah, AlQurayat, and Riyadh reported multiple outbreaks in more than 10 cities and districts. The highest incidence was reported in the AlJouf region in 2019, with 780 cases in five cities, followed by 286, 242, 230, 48, and 31 in AlGurayat, Tabuk, AlQassim, Riyadh, and Jeddah regions, respectively, with an average age of six (±2) years [5-10]. Omer et al. reported that 95.5% of individuals were non-vaccinated, whereas other studies revealed that unvaccinated individuals accounted for approximately one-third of the cases [5-7,9,10]. In addition, they stated that among the affected children, 4.2% required admission to the pediatric intensive care unit (PICU) due to severe complications such as measles-related pneumonia [6]. AlAbdullah et al. reported that among 31 patients, 50% required hospitalization, with one patient admitted to the ICU due to pneumonia. No deaths were recorded, indicating the effectiveness of rapid intervention and appropriate treatment [9].

Historically, Saudi Arabia has attempted multiple modifications to the vaccination schedule to aid in measles elimination, with the last revision done in 2013 [8,11]. The program called for the initial measles-containing-vaccine first dose (MCV1) to be administered at nine months, followed by the MMR dose at 12 and 18 months, and concluding with the last MMR dose for children aged four to six years. Since then, the measles incidence rate had not been of concern until the end of 2022 when a small community outbreak was noted mainly among refugees. Moreover, due to quarantine and disruption of vaccination campaigns, the SARS-CoV-2 pandemic may result in a faster outbreak of measles worldwide [12,13].

The number of measles cases has been increasing in Saudi Arabia since January 2023. Therefore, we conducted an outbreak investigation to describe the epidemiology, clinical characteristics, and outcomes of Measles infection among children at King Abdullah Specialized Children’s Hospital (KASCH), a tertiary and academic free-standing hospital.

## Materials and methods

KASCH is a tertiary hospital in the east of Riyadh City in Saudi Arabia. The hospital’s infection control unit was notified about the first case of measles infection on January 15, 2023. Measles infection is classified as a class-1 reportable disease in Saudi Arabia. All hospitals must report any suspected or confirmed measles cases through the infection control department to the Ministry of Health through the Healthcare Electronic Surveillance Network for outbreak control measures. The case definition according to the WHO criteria for measles infection was followed, which is based on clinical and/or laboratory evidence of infection. Clinical findings include fever, maculopapular rash, and at least one symptom of cough, coryza, or conjunctivitis. Laboratory confirmation is achieved by the detection of anti-measles IgM antibody, a positive reverse transcription-polymerase chain reaction, viral isolation in cell culture, or a four-fold increase in anti-measles IgG antibody.

We included all pediatric patients with confirmed measles infection by a detectable anti-IgM antibody level through the identification system within the existing hospital infection control database from January 15, 2023, through March 15, 2023, with no exclusion criteria. A descriptive cross-sectional study design was implemented. Electronic medical records and direct chart review were employed to gather relevant data, including demographic characteristics, clinical presentation, and clinical outcomes. Vaccination information was extracted from the patient’s medical record, immunization card, or by contacting primary care providers. Data confidentiality and privacy were ensured throughout the study period. All data collection tools were anonymous.

Statistical analysis

Descriptive statistics are reported for demographic variables, clinical presentation, interventions, and complications of patients with confirmed measles using frequencies for categorical variables and mean and standard deviation for continuous variables. Moreover, the prevalence of different age categories among confirmed measles patients in intensive care admissions, days between rash appearance and Koplik’s spots, vaccination status by anti-measles IgG antibody, and antibiotic administration due to complications among confirmed cases were examined using chi-square tests, indicating if those prevalence rates were significantly different. All Analyses were done using SPSS (IBM Corp., Armonk, NY, USA).

## Results

**Figure 1:**
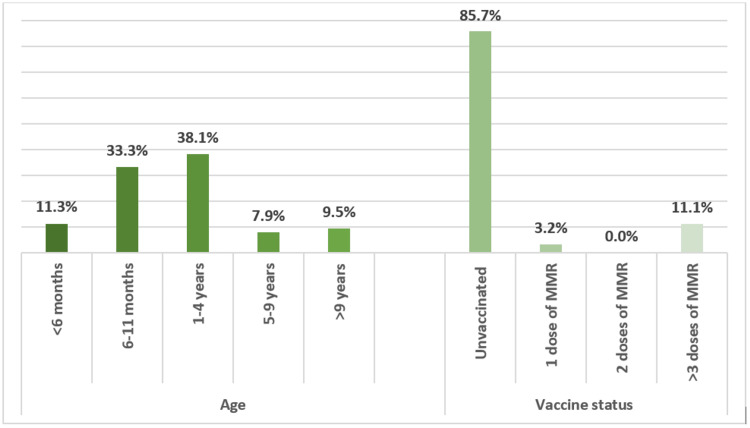
Age distribution in measles cases and vaccination status.

A total of 63 confirmed cases of measles were reported. Table 1 shows the demographic characteristics. There was an almost equal gender distribution, with 55.6% males and 44.4% females. In addition, a similar equal incidence rate was noted between the Saudi nationality (47.6%) compared to others (52.4%). Figure 1 shows the age distribution and vaccination status of measles cases. Overall, 82.7% of the cases were under the age of four years, and 95.2% were healthy. Further, 85.7% of confirmed cases were found to be unvaccinated, and 11.1% of individuals had received more than three doses of MCV.

**Table 1:** Demographic characteristics of measles patients (n = 63). *: Hydrocephalus, dysmorphic, and cerebral palsy; **: Bronchopulmonary dysplasia and preterm. CNS: central nervous system

Characteristic	N (%)
Sex	
Male	35 (55.6)
Female	28 (44.4)
Nationality	
Saudi	30 (47.6)
Non-Saudi	33 (52.4)
Underlying disease	
Healthy	60 (95.2)
Syndromic/CNS*	2 (3.2)
Respiratory**	1 (1.6)

Classical measles manifestations were reported in the vast majority of the patients, with fever and rash (100%) recorded more frequently in young children, followed by cough (87.3%), conjunctivitis (57.1%), diarrhea (54%), Koplik’s spots (31.7%), and vomiting (31.7%). Other less common presentations were lymphadenopathy, strawberry tongue, and syncope in 9.5%, 4.8%, and 1.6% of patients, respectively. In addition, 80% of Koplik’s spots were documented from the first day until the third day of the rash, while 20% persisted up to seven days from the onset of the rash.

**Figure 2:**
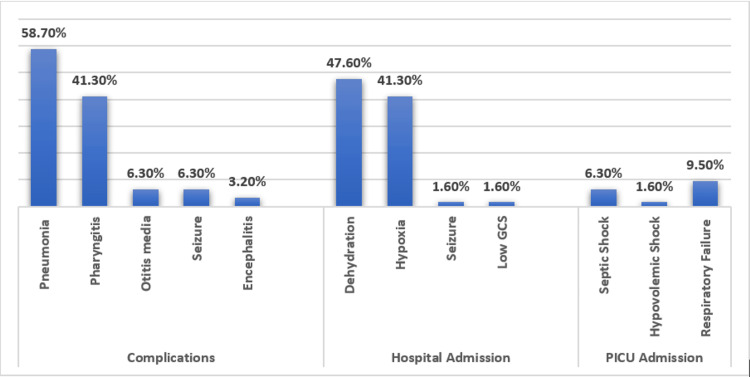
Measles complications and reasons for admission. PICU: pediatric intensive care unit

Table 2 and Figure 2 present a summary of the clinical characteristics, complications, and therapy provided for patients admitted as a case of measles. Overall, 30% of patients had viral coinfections, with adenovirus reported in 12.7%, followed by rhino/enteroviruses in 7.9%, and parainfluenza in 4.8%. Other viruses were also reported, including human coronavirus (1.6%) and influenza (1.6%). Among the observed chest X-ray findings, 20.6% showed bilateral bronchial wall thickening, 19% presented with a localized right parenchymal infiltrate, and 15.9% had a patchy bilateral infiltrate. Most children (64.5%) received antimicrobials, with ceftriaxone being the most frequently used antibiotic (53.2%). Overall, 48% of our cases used antibiotics for the treatment of pneumonia with a significant p-value (<0.001), followed by 26% and 7% treating pharyngitis and otitis media, respectively. The vast majority of our cases received vitamin A therapy for their illness (82.5%). Intravenous immunoglobulin was given in only one case, and steroid therapy was given to six of our patients.

**Table 2:** Investigation and interventions among measles patients (n = 63). PICU: pediatric intensive care unit

Variable	N (%)
Positive viral test	
Rhino/enterovirus	5 (7.9)
Adenovirus	8 (12.7)
Parainfluenza	3 (4.8)
Coronavirus	1 (1.6)
Influenza	1 (1.6)
Chest X-ray finding	
Bilateral bronchial wall thickening	13 (20.6)
Right parenchymal infiltrate	12 (19)
Bilateral patchy infiltrate	10 (15.9)
Pleural effusion	2 (3.2)
Number of admissions	56 (88.9)
Duration of admission (days), mean ± SD	4.5 ± 5.8
Number of PICU admission	11 (17.5)
Duration of PICU admission (days), mean ± SD	7.1 ± 7.6
Antibiotics administration	
Received at least one antibiotic	40 (64.5)
Ceftriaxone	33 (53.2)
Azithromycin	2 (3.2)
Amoxicillin	10 (16.1)
Vancomycin	7 (11.2)
Vitamin A administration	52 (82.5)
Outcomes	
Death	1 (1.6)
Discharge	62 (98.4)

Unusual presentation of complicated pneumonia with pleural effusion was seen in 3.2% of cases. Out of the total cases, approximately 58.7% developed pneumonia, and 88.9% were hospitalized. The main reasons for hospitalization were the need for intravenous fluids (47.6%) and oxygen (41.3%). Within the hospitalized group, 17.5% required PICU admission, primarily due to respiratory failure (9.5%) and septic shock (6.3%).

There were significant differences in the vaccination status according to the level of anti-measles IgG antibody (immunity status) (p < 0.05) (Table 3).

**Table 3:** Prevalence of vaccination status by measles IgG value among measles patients (n = 63). *: Measles IgG level less than 16.5 AU/mL.

Vaccination status	Measles IgG, N (%)		P-value
	Non-immune*	Immune	
Unvaccinated before exposure	47 (87)	7 (13)	0.001
Received the first dose	2 (100)	0 (0)	
Received more than three doses	2 (28.6)	5 (71.4)	

## Discussion

In our study, an outbreak of measles surged in January in an entire apartment building in Riyadh occupied by unvaccinated Syrian refugees. At first, most cases were Syrian; later, when the disease spread rapidly, a similar incidence rate was noted among Saudis and those of other nationalities. This cross-sectional study showed that 82.7% of children with measles were under four years of age, and the majority of cases were not vaccinated (85.7%). Pneumonia was the most common complication, leading to most children being hospitalized secondary to hypoxia. In addition, respiratory failure was the most common cause of PICU admission, especially in children under the age of one year. Most of our patients had the classic clinical presentation of measles, including fever, rash, conjunctivitis, cough, pharyngitis, lymphadenitis, and arthralgia.

In this study, Koplik’s spots were observed in one-third of patients. These are small white lesions, approximately 2-3 mm in diameter, with an erythematous base on the buccal mucosa. They appear one day before the onset of the rash and last for two to three days after their appearance and are considered a pathognomonic feature of measles [14]. Further, we found that 20% of these spots persisted for up to seven days without any significant clinical correlation with the severity of the disease.

A significant difference in measles infection in regards to signs and symptoms was not observed between the two genders, in alignment with previous reports [5,15]. Most cases of measles in our cohort were under the age of four, accounting for 82.7% of cases, consistent with the Saudi surveillance data and most of the previously reported local and international outbreaks [5,15-18]. Older children had a lower incidence rate due to higher immunization status before the pandemic. In our study, 85.7% were unvaccinated, with the majority attributed secondary to the SARS-CoV-2 pandemic or lack of health awareness among refugee families. Further breakdown of the age revealed that 44.6% were infants under one year of age, while 38.1% were children between one and four years of age. However, 11.3% were unvaccinated due to the limited age of under six months. This comparable rate between the two groups could be explained by the lack of vaccination for age-eligible children. On the other hand, the high rate in infants younger than one year is believed to be secondary to unvaccinated mothers or mothers with low IgG levels to measles, as maternal antibodies are fundamental for an infant’s immunity [19-21].

According to the Advisory Committee on Immunization Practices, specific criteria are considered for presumptive evidence of immunity to measles, including documented reception of age-appropriate live measles virus vaccine and laboratory evidence of immunity or disease or birth date before 1957 [22]. The efficacy of the MMR vaccine in preventing measles is well established, and immunity is achieved after two doses of MMR. However, the emergence of measles outbreaks among immunized groups can be attributed to vaccine failure (primary failure), waned immunity (secondary failure), or the degree of exposure [23]. Our study found that 11.1% of infected children received three doses of measles vaccine, while 71.4% had a good level of anti-measles IgG antibody. Similar measles cases have been reported in previously vaccinated individuals with a detectable anti-measles IgG antibody level [24,25].

Several studies have shown that infected immune patients are asymptomatic, have mild symptoms, or have a shorter hospital stay [23-27]. Similar findings have been found in our study. Most patients who presented with clinical signs and symptoms without complications were admitted as a consequence of dehydration and were discharged within one to three days of admission.

Measles can have serious complications, particularly in young children, adults over 20 years old, pregnant women, and those with weakened immune systems or malnutrition. The respiratory and digestive systems are most commonly affected, with pneumonia and ear infections being the most commonly reported infections [28]. Our findings are consistent with previous reports, as the majority of our measles cases had upper respiratory tract infections.

Measles can also lead to serious neurological complications and a high risk of severe sequelae, including death [29,30]. Primary measles encephalitis is a rare condition that was more prevalent in the past, as measles cases have decreased significantly in recent decades. A study conducted between 1947 and 1957 on 61 patients with measles encephalitis found that only about one in 1,000 cases resulted in clinical encephalitis. Additionally, the study showed that half of the patients were between the ages of five and seven. Unfortunately, the mortality rate for this condition is 10-15%, and 25% of patients experience permanent neurological damage [30]. During this outbreak, we reported two cases of encephalitis in younger immunocompetent individuals, with no fatalities. The first case presented with a transient state of encephalitis in an unvaccinated two-year-old Syrian refugee who presented with fever, rash, vomiting, and diarrhea along with a decreased level of consciousness and a history of measles exposure. After a few days of supportive therapy, his neurological status improved. The other severe case of encephalitis was seen in a five-year-old unvaccinated Syrian refugee who presented with rash, fever, focal seizure, and altered level of consciousness, followed by right-sided hemiparesis and slurred speech. He was admitted to the PICU secondary to septic shock. His brain imaging showed unilateral, multifocal, left-sided, mainly cortical and deep nuclei, abnormal high signal intensities with corresponding restricted diffusion with no definite enhancement. His recovery time was prolonged (one month) with no medical intervention.

Bacterial or viral coinfections have been associated with increasing disease severity and complications. Viral coinfection with measles has been reported since 1998 [31]. Several studies revealed that adenovirus and parainfluenza were the most frequently reported [31,32]. In one study, adenovirus incidence was reported to be 19%, and parainfluenza incidence was reported to be 25% [31]. In other studies, adenovirus was detected at a higher incidence (24.1%), followed by respiratory syncytial virus (11.1%) and recombinant human parainfluenza virus type 3 (3.7%). One-third of our patients had been coinfected with other viruses. Adenovirus was predominant at 12.7%, followed by rhino/enterovirus and parainfluenza at 7.9% and 4.8%, respectively.

During a 2014 measles outbreak in Vietnam, postmortem pathologic examination of hospitalized children who died showed that pneumonia due to adenovirus type 7 was a contributory cause of death in children with measles-associated immune suppression [33]. Antimicrobial therapy has been used to treat bacterial coinfection in previous outbreaks [34]. More than half of our patients received antibiotics primarily for pneumonia. According to one systematic review, antibiotics given to children with measles decrease the risk of otitis media and tonsillitis and reduce the likelihood of pneumonia [35]. However, prophylactic antibiotics are discouraged due to the substantial risk of antimicrobial resistance.

In this study, the hospitalization rate was relatively high at 88.9%. It is worth noting that rates of hospitalization as low as 20% or as high as 70% have been observed in other studies [36-39]. We found that children under four years old were determined to be three times more likely to be hospitalized than older children. Additionally, these children may experience significant complications that demand additional medical attention. The majority of our patients suffered from considerable dehydration that necessitated the administration of intravenous fluids (47.6%) or pneumonia requiring oxygen therapy (41.3%), which closely aligns with findings from other studies [6,34]. A significant proportion of children (17.5%) required admission to the PICU compared to other studies (1.9-4.2%). It was primarily due to respiratory failure and, subsequently, septic or hypovolemic shock [6,38]. Furthermore, admission to the PICU was strongly linked to children less than one year of age.

The majority of children in our cohort were able to return home in good health, with only one reported death (1.6%). The hospital course of this patient was complicated by septic shock and respiratory failure. He had been vaccinated with good anti-measles IgG antibody level. However, he had a secondary bacterial infection with *Klebsiella pneumoniae* urinary tract infection. The rate of measles-related deaths is lower in this study than previous studies have reported (3.3%-9%). This could be because no underlying complex chronic conditions or malnutrition were present, which are typically the leading causes of morbidity and mortality associated with measles [14,40-43]. Furthermore, our patients received prompt and adequate care, highlighting the importance of early diagnosis and treatment in preventing complications and reducing mortality rates, along with the easy accessibility of healthcare centers where free treatment is provided [43]. Complications such as bronchopneumonia or encephalitis (one of the leading causes of death in developing countries) often contribute to deaths from measles [6,41-44]. In the PICU, hospitalization lasted an average of 7.1 days compared to 4.5 days in the general ward, consistent with previous reports [34,36,45].

This study has several limitations. Our study did not include individuals who did not seek medical care, asymptomatic patients, or those discharged from the emergency department without serological testing. Furthermore, we did not evaluate factors that could contribute to incomplete immunization, such as socioeconomic status or barriers to accessing healthcare facilities. Finally, this study was conducted in the largest pediatric center in Saudi Arabia. Further multi-center studies are required to represent accurate epidemiological data and prevent future outbreaks.

## Conclusions

In addition to the substantial health consequences and financial burdens that result from measles, the recent outbreak of the disease serves as a reminder that vaccination and herd immunity are crucial. Moreover, it highlights the potential repercussions of not vaccinating and affecting public health, as well as the need for ongoing vigilance and proactive measures to prevent future outbreaks.
